# Dose-volumetric parameters and prediction of severe acute esophagitis in patients with locally-advanced non small-cell lung cancer treated with neoadjuvant concurrent hyperfractionated-accelerated chemoradiotherapy

**DOI:** 10.1186/1748-717X-8-122

**Published:** 2013-05-17

**Authors:** Farkhad Manapov, Susanna Sepe, Maximilian Niyazi, Claus Belka, Godehard Friedel, Wilfried Budach

**Affiliations:** 1 Klinik und Poliklinik für Strahlentherapie und Radioonkologie, Klinikum der Universität München, München, Germany; 2 Klinik für Thoraxchirurgie; Robert-Bosch-Hospital, Klinik Schillerhöhe, Stuttgart-Gerlingen, Germany; 3 Klinik für Strahlentherapie und Radioonkologie, Universitätsklinikum Düsseldorf, Heinrich Heine Universität Düsseldorf, Düsseldorf, Germany

**Keywords:** Esophagitis, Hyperfractionated accelerated chemoradiotherapy, Lung cancer

## Abstract

**Background:**

To identify dose-volume parameters predictive for severity of acute esophagitis (CTC > grade 2) in locally-advanced non small-cell lung cancer (LA-NSCLC) patients treated with neoadjuvant concurrent hyperfractionated-accelerated chemoradiotherapy (HA-CRT) a retrospective analysis was performed. 88 patients were treated with HA-CRT followed by radical surgery. Predictive power of absolute oesophageal length, absolute and relative oesophageal volume included in the 95%-isodose, patient- and tumor-related factors for severity of acute esophagitis was assessed.

**Findings:**

A total of 82 patients (93%) developed radiation-induced acute esophagitis. Grade 1 was documented in 1 (1%), grade 2 in 55 (67%), grade 3 in 23 (28%) and grade 4 in 3 (4%) patients, respectively. Absolute oesophageal volume included in the 95%-isodose (42.8 Gy) achieved 13.5 cm^3^ (range: 3 – 29 cm^3^). Of the tested variables in univariate analysis, absolute oesophageal volume included in the 95%-Isodose was found to be the only significant variable (p = 0.03) predicting severe acute esophagitis (CTC > grade 2). For this volume a gradation scale of the likelihood of severity was built.

**Conclusion:**

Increase of absolute oesophageal volume included in the 95%-isodose correlates with severity of acute esophagitis in LA-NSCLC patients treated with neo-adjuvant concurrent HA-CRT.

## Background

Concurrent chemoradiotherapy (CRT) is the most relevant treatment option for inoperable LA-NSCLC. Meta-analysis documented that definitive concurrent CRT is associated with a significant prolongation of overall survival compared to thoracic irradiation (TRT) alone [1]. However, local control is still poor. Thus, new treatment protocols aiming to increase local control are urgently needed. Increasing the dose density via hyper-fractionated-accelerated radiation protocols represents one of the known options in order to increase local control rates. Saunders et al. were the first who documented a superiority of HA-radiotherapy over conventional treatment regarding local relapse and survival in NSCLC [2]. Similar results were reported for limited-disease small-cell lung cancer treated with CRT [3]. RTOG 9410 phase III randomised trial for LA-NSCLC which compared three different definitive CRT regimens has reported that there were significantly fewer patients with disease progression within the radiation field when treated with concurrent HA-CRT [4].

An even more intense treatment in implemented when a concurrent application of HA-TRT and chemotherapy is followed by radical resection. At least two single-centre trials have applied such a protocol and demonstrated up to 60% of pathological down-staging and significantly improved long-term survival [5-7]. A multicentre randomised phase III trial investigating this tri-modality concept is still ongoing (EsPaTü) [8].

As trade-off, any dose escalation and treatment intensification of TRT generally results in higher toxicity. Besides pneumonitis, acute esophagitis is a highly important adverse effect and represents a dose-limiting toxicity [9]. Therefore, the identification of predictive factors for severe radiation-induced acute esophagitis is of high clinical relevance. Most data concerning this clinical problem reflect the situation in patients treated with conventional TRT and it has to be questioned in how far these data can be applied to HA-CRT. To define relevant parameters in patients with hyper-fractionated tri-modality concepts the impact of several dose-volume parameters, patient- and tumor-related factors on the severity of acute oesophageal reaction in patients with LA-NSCLC were analysed.

## Findings

All 88 patients (median age 55 years for women and 58 years for men) have participated in a prospective phase II trial [5,7], were diagnosed with initially inoperable histologically proven LA-NSCLC and treated with neoadjuvant HA-CRT followed by radical surgery whenever possible. All patients had a performance score (PS) WHO 1–2. LA-NSCLC was defined as a disease corresponding to Stage III A(2)-B after completion of initial staging. All patients provided written informed consent.

Patients received four cycles of induction chemotherapy (weekly carboplatin dosed at AUC 2 and paclitaxel 100 mg/m^2^) followed by 3 weeks of HA-CRT with 1.5 Gy twice a day up to 45 Gy combined with weekly carboplatin AUC 2 and paclitaxel 50 mg/m^2^. Irradiation was delivered with a linear accelerator using a multiple field technique. 3D-CT treatment planning was performed. Clinical target volume included primary tumor with 1.0-cm safety margin and bilateral mediastinal nodes from the jugulum to the level of the heart valves. Lower mediastinum was included if nodal involvement was suspected. For each patient oesophagus was delineated from cricoid to gastro-oesophageal junction. Total absolute oesophageal length and volume as well as length and volume included in the 95%-isodose (42.8 Gy) were measured. Dose-volume histograms of oesophagus were provided for each individual patient. Symptom assessment of acute esophagitis was performed weekly during the whole course of HA-CRT. Severity of acute esophagitis was documented according to the “CTC v. 2”. Putative correlations between absolute total oesophageal length, length included in the 95%-isodose, absolute total oesophageal volume, volume included in the 95%-Isodose and relative oesophageal volume within the 95%-Isodose as well as age at diagnosis, gender, initial T-stage, hematotoxicity and severity of the acute oesophageal reaction were analysed using the binary logistic regression module of the “XLSTAT” software package. *P*-values and asymptotic confidence limits were calculated based on the respective co-variance matrices.

A total of 82 treated patients (93%) developed acute esophagitis. Grade 1 acute esophagitis was detected in 1 (1%), grade 2 in 55 (67%), grade 3 in 23 (28%) and grade 4 in 3 (4%) patients, respectively. All 82 patients received symptomatic care. No esophagogastroduodenoscopy was necessary during the whole course of treatment. Dose-volumetric parameters of oesophagus were correlated to the corresponding CTC-degree of the radiation-induced acute esophagitis for each individual patient. A similar analysis was done for patient- and tumor-related factors.

**Figure 1:**
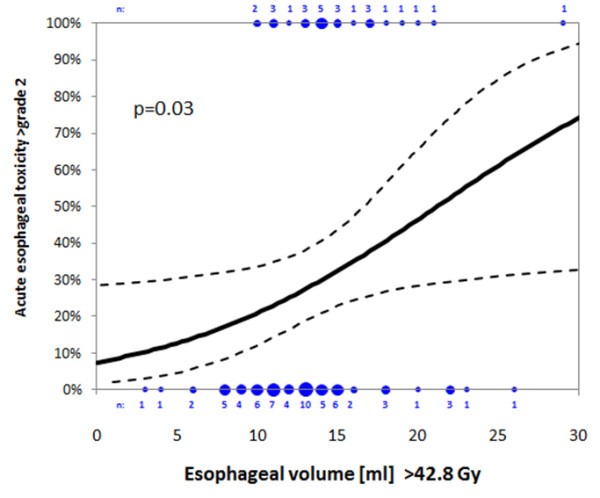
**Likelihood of acute severe acute esophageal reaction (>CTC grade 2) depends on the absolute esophageal volume within 95%-Isodose (>42.8 Gy).** The original binary data and the resulting binary logistic regression line including the 95% confidence limits (dashed lines) are displayed. Blue symbols and numbers indicate events and number of events at the respective volume.

On average, absolute total oesophageal length was 23.5 cm (range: 18 – 28 cm) and median length included in the 95%-isodose (42.8 Gy) reached 13 cm (range: 6 – 21 cm). Median total oesophageal volume was 24.5 cm^3^ (range: 9 – 43 cm^3^). Volume included in the 95%-isodose (42.8 Gy) achieved on average 13.5 cm^3^ (range: 3 – 29 cm^3^) and accounted for 51% (range: 18 – 89%) of total organ volume. Of the tested variables in the univariate analysis, absolute oesophageal volume included in the 95%-isodose was found to be the only significant variable (p = 0.03) predicting severe acute esophagitis (CTC > grade 2). No events of severe esophagitis were observed at oesophageal volumes in the 95%-isodose below 10 ml. The original data and the resulting logistic regression are shown in Figure 1.

No significant correlation for patient- and tumor-related factors and severity of radiation-induced acute esophagitis was found.

## Discussion

Neoadjuvant concurrent HA-CRT followed by radical surgery in LA-NSCLC was shown to be a promising approach in single-centre studies and a multi-institutional randomized phase III trial is actually ongoing [5-8].

In previous trials dose escalation and intensification of TRT resulted in higher acute oesophageal toxicity which was established as dose-limiting in patients treated with different types of CRT. RTOG 9410 trial investigating three different regimens reported a 45% rate of grade 3 acute esophagitis in the accelerated CRT arm [4]. Earlier, Ball et al. have shown a significant increase of the rate of severe acute esophagitis from 21% in the conventional on 42% in accelerated CRT arm [10]. RTOG database analysis on 528 LA-NSCLC patients revealed a strong association of severe acute esophagitis with hyperfractionated CRT [11].

That is why a question arises again whether the severity of acute esophagitis can be predicted on the basis of individual dose-volumetric parameters. Patel et al. reported in accelerated CRT that the critical parameter for development of acute esophagitis was the oesophageal volume treated with > 50 Gy [12]. A japanese study of conventional CRT from Hirota and colleagues recommended that the absolute oesophageal length and oesophageal volume within 45 Gy-Isodose should amount less than 9.5 cm and 40%, respectively [13]. Importantly, both studies included only a small number of patients. De Ruyck et al. have found mean oesophageal dose, overall treatment time and radiation technique as important parameters predicting severe acute esophagitis [14].

Present study analysed highest number of patients treated with neoadjuvant concurrent HA-CRT. Our analysis was built on the investigation of individual TRT plans. Importantly, there were no differences in regard to the PS, TNM stage and applied multimodality protocol within the treated population. A comparison of calculated dose-volumetric parameters with carefully assessed individual symptoms of acute esophagitis during the HA-CRT was a basis for our investigation. An absolute oesophageal volume received a dose of at least 42.8 Gy (within the 95%-isodose) was identified as a dose-volumetric predictor for the severity of acute oesophageal reaction in treated population. The increase of this volume was strongly correlated with corresponding increase of the CTC-grade and a gradation scale of the likelihood of severity of acute esophagitis was created. Results of our study gain special importance in the scope of the ongoing multicentre randomized phase III trial. Intensification of a neoadjuvant approach due to combination of the chemo- and radiation therapy defines an actual trend in the multimodality treatment of LA-NSCLC. Prediction of the severity of acute esophagitis based on individual dose-volumetric parameters help to complete overall treatment without interruption. Our gradation scale built a tool for further optimization of radiation therapy treatment planning in LA-NSCLC.

## Competing interests

All authors declare that they have no competing interests.

## Authors’ contributions

All authors have made substantial contributions. There is no financial relationship between the authors and commercial companies whose products were used in the study. All authors read and approved the final manuscript.
